# Clade-3 Bat Sarbecovirus PRD0038 Reveals Constraints on Coronavirus Emergence and Immune Sensitivity

**DOI:** 10.21203/rs.3.rs-9954172/v1

**Published:** 2026-07-02

**Authors:** Anfal Abdelgadir, Edgar F. Kong, Ruth J. Parsons, Michael L. Mallory, Boyd L. Yount, Trevor Scobey, John M. Powers, Kendra L. Gully, Lily E. Adams, Robert J. Edwards, Katayoun Mansouri, Priyanka Devkota, Katarzyna Janowska, Rasangi Pathirage, Nicholas J. Catanzaro, Abbey L. Biggers, Jason J. Lavinder, Gregory C. Ippolito, Mark T. Heise, Priyamvada Acharya, Lisa E. Gralinski, Ralph S. Baric

**Affiliations:** 1. Department of Microbiology and Immunology, University of North Carolina at Chapel Hill, Chapel Hill, NC, USA; 2. Department of Epidemiology, University of North Carolina at Chapel Hill, Chapel Hill, NC, USA; 3. Duke Human Vaccine Institute, Durham, NC, USA; 4. Duke University, Department of Biochemistry, Durham, NC, USA; 5. Duke University, Department of Medicine, Durham, NC, USA; 6. Department of Chemical Engineering, The University of Texas at Austin, Austin, TX, USA; 7. Texas Biomedical Research Institute, San Antonio, TX, USA; 8. Department of Genetics, University of North Carolina at Chapel Hill, Chapel Hill, NC, USA; 9. Duke University, Department of Surgery, Durham, NC, USA

**Keywords:** PRD0038, Sarbecovirus, Coronavirus, Spike, epidemiology, antiviral

## Abstract

Zoonotic Sarbecoviruses present a documented threat to global health. Here, we report the recovery of an African clade-3 recombinant Sarbecovirus, PRD0038, and derivatives encoding reporter genes. Cryo-EM structural analyses of rPRD0038 revealed spike glycoprotein sites that facilitate the receptor-binding domain (RBD)-up conformation, enhancing replication in *R. affinis* but not human ACE2 expressing cells. Host range analysis of rPRD0038 identified civets, rabbits, camels, and cows as potential intermediate hosts, while confirming the lack of human ACE2 usage and replication in primary human airway epithelial cells. Despite significant divergence from SARS-CoV-2, PRD0038 remains susceptible to FDA-approved nucleoside and mPro-targeted antivirals as well as some monoclonal antibodies that target select conserved spike epitopes. Pre-existing SARS-CoV-2 immune memory conferred reduced cross-neutralizing activity against PRD0038. Finally, we present an *R. affinis* ACE2-expressing mouse model for assessing PRD0038 *in vivo* replication and pathogenesis, and testing countermeasures. Together, these data illustrate functional and immunological constraints that regulate the emergence potential of clade-3 bat Sarbecoviruses while identifying protective therapeutics.

## INTRODUCTION

The emergence of SARS-CoV-2, which caused the COVID-19 pandemic, caused over 770 million infections and more than 7 million deaths globally^1–3^. Sarbecoviruses, including SARS-CoV and SARS-CoV-2, are zoonotic pathogens, and identifying their natural reservoirs and potential intermediate hosts is critical for containment, preparedness, and prevention of future outbreaks^4,5^. Global surveillance has established bats, particularly *Rhinolophus sp*., as the principal reservoirs for Sarbecoviruses with human emergence potential, while also revealing the extensive viral genetic and antigenic diversity^6,7^. High RNA recombination rates, coupled with broad receptor usage, provide novel pathways for Sarbecovirus evolution and immunologic escape^8^. African Sarbecoviruses, while abundant, remain poorly characterized compared to Asian strains, leaving a significant gap in our understanding of this high-risk Sarbecovirus clade.

Recently, *Rhinolophus sp*. bats sampling in Rwanda led to the identification of a novel clade-3 Sarbecovirus, designated PRD0038. This virus shares ~76% and 74% nucleotide identity with SARS-CoV and SARS-CoV-2, respectively^9^. PRD0038 receptor-binding domain (RBD) structural modeling shows overall similarity to SARS-CoV and SARS-CoV-2, but substitutions at key interface residues prevent efficient binding to human ACE2^9,10^. Biolayer interferometry and pseudovirus assays confirmed recognition of *Rhinolophus* ACE2, including *R. alcyone*, *R. affinis*, *R. sinicus*, and *R. landeri*. In pseudovirus assays, a single substitution (T487W) or the combined substitutions K482Y/T487W partially restored human ACE2 usage, underscoring the zoonotic potential of this clade^10^.

Structural models of PRD0038 suggest that the spike adopts a closed RBD conformation^10^. However, the impact of this configuration on viral entry, replication, and host tropism remains unknown. In SARS-CoV-2, the D614G substitution, which emerged early in the pandemic, shifts the spike toward an RBD-up conformation, enhancing virion infectivity and transmissibility^11–14^. Whether analogous substitutions in zoonotic Sarbecoviruses similarly influence spike dynamics and receptor accessibility remains poorly studied. Additionally, fundamental aspects of PRD0038 biology remain unexplored, including live virus growth, receptor tropism in non-bat mammals, and *in vivo* replication, pathogenesis, and susceptibility to existing countermeasures.

Here, we provide a comprehensive characterization of a recombinant rPRD0038, as a prototype clade-3 Sarbecovirus recovered in cell culture. Using live reporter viruses and novel cell lines expressing diverse mammalian ACE2 orthologs, we identify potential spillover hosts and confirm entry-level restrictions for human receptors. We identify natural sites that regulate rPRD0038 RBD presentation and replication in bat ACE2 cells, while confirming the lack of replication in primary and continuous cells expressing human ACE2. We further assess the susceptibility of rPRD0038 to FDA-approved antivirals, available neutralizing monoclonal antibodies, and preimmune sera from SARS-CoV-2 vaccinated humans and non-human primates. As PRD0038 and other bat Sarbecoviruses cannot use human, mouse, or hamster ACE2 receptors for entry, we developed and tested a transgenic *R. affinis*-ACE2 mouse model to evaluate the efficacy of available countermeasures against bat Sarbecoviruses. Collectively, our findings inform surveillance strategies, vaccine design, and preparedness against emerging Sarbecoviruses with high pandemic potential.

## RESULTS

### Phylogenetic placement and reverse genetic recovery of PRD0038

**Extended Data Fig. 1:**
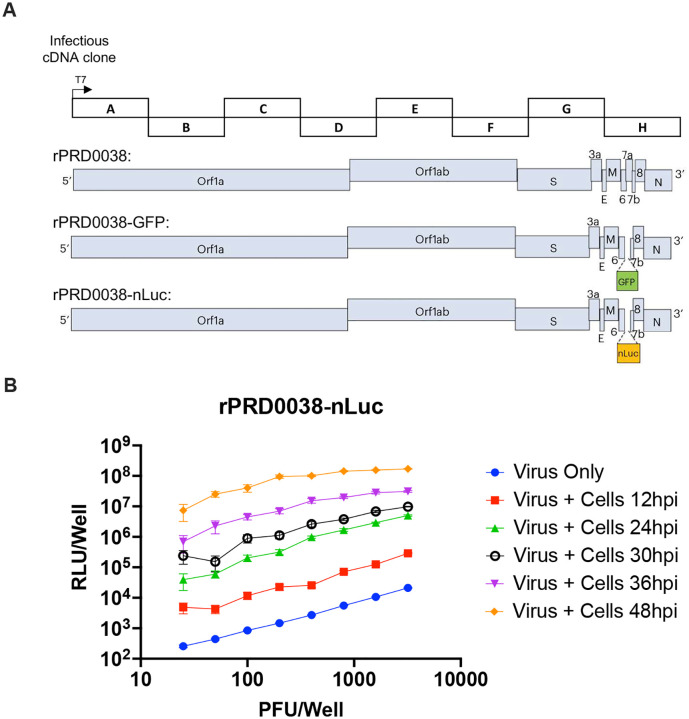
Schematic design and growth curve analysis of reporter rPRD0038 variants. **A**. Genome schematic of rPRD0038 infectious cDNA clone as well as green fluorescent protein (GFP) and nano-luciferase (nLuc) reporter variants. **B.** Infection kinetics of the rPRD0038-nLuc reporter virus. Cells were infected with the indicated PFU/well, and nLuc activity was measured at 12, 24, 30, 36, and 48 hours post-infection (hpi). Data are mean ± s.e.m. of a single experiment performed in duplicate.

**Figure 1:**
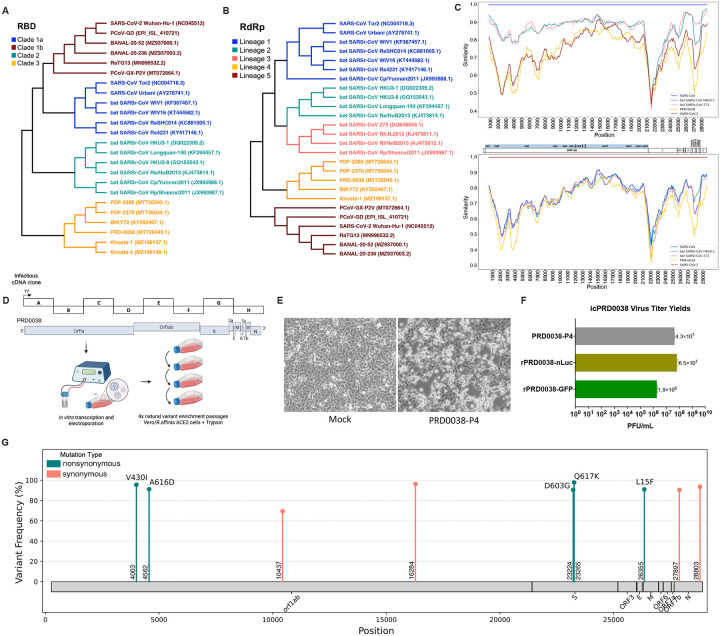
Recovery and characterization of recombinant PRD0038. **A**. Phylogenetic tree on the amino acid sequence of the Receptor Binding Domain (RBD) of the spike protein of sarbecoviruses. Colors represent the different clades (clade1a in blue, clade 1b in brown, clade 2 in green, and clade 3 in yellow). **B.** Phylogenetic tree of the RNA-dependent RNA polymerase (RdRp) gene (nsp12) for sarbecoviruses. Colors of clade bars represent the different lineages (lineage 1 in blue, lineage 2 in green, lineage 3 in salmon, lineage 4 in yellow, and lineage 5 in brown). **C.** Similarity plot analysis of representative sarbecoviruses from each lineage based on the full-length genome sequence of the SARS-CoV (top) or SARS-CoV-2 (bottom) as a reference. The analysis was performed using a Python-based implementation of SimPlot (SimPlot++, window size = 200bp, step size = 30bp). **D.** Schematic of the PRD0038 infectious cDNA clone design and subsequent *in vitro* RNA transcription, electroporation, and natural quasispecies variant enrichment by passaging in the in Vero cells expressing *R. affinis* ACE2 with 5 μg/mL trypsin. **E.** Cytopathic effect (CPE) of the PRD0038 passage 4 (P4) observed on Vero/*R.affinis* ACE2 cells 72hpi. **F.** Virus titer measured by plaque assay as PFU/mL for PRD0038-P4 and two recombinant strains encoding GFP and nLuc reporter genes, and nonsynonymous substitutions accumulating in natural variant enrichment. Data represent the mean ± s.e.m of two independent experiments. **G.** Lollipop plot showing the distribution of synonymous (salmon) and nonsynonymous (green) substitutions across the PRD0038 genome identified by next-generation sequencing of PRD0038-P4 following natural quasispecies variant enrichment. Each vertical line indicates a mutation position, with stem height proportional to variant frequency. Coding regions are annotated along the x-axis.

Phylogenetic analysis of the RBD places PRD0038 in a separate clade (clade 3), distinct from SARS-CoV (clade 1a), SARS-CoV-2 (clade 1b), and SARS-related bat viruses (clade 2) (Fig. 1A). Analysis of the RNA-dependent RNA polymerase (RdRP) reveals five major Sarbecovirus lineages, with the Rwanda-derived PRD0038 forming a new lineage 4 alongside other African bat Sarbecoviruses (Fig. 1B). PRD0038 shares approximately 76.5% genome-wide nucleotide identity with SARS-CoV and about 74.5% with SARS-CoV-2, with significant divergence observed around nsp2, nsp3, and the spike protein (Fig. 1C). As such, PRD0038 represents a diverse, understudied Sarbecovirus with unknown emergence potential. To characterize PRD0038 replication and growth kinetics, we generated a cDNA clone from the full-length virus sequence^9^, followed by *in vitro* transcription using T7-RNA polymerase and electroporation into Vero81 cells expressing *R. affinis* ACE2 (Fig. 1D). Cytopathic effects were detected in Vero/*R.affinis* ACE2 cells 72 hours post-infection with PRD0038 stocks derived from virus growth under low-trypsin conditions (Fig. 1E). Titers of the generated working stock (P4) reached 4.3 × 10^7^ PFU/mL, in the absence of trypsin treatment (Fig. 1F). Next-generation sequencing of the P3 and P4 virus stocks, compared to the published reference genome, revealed the accumulation of naturally occurring quasispecies variants, including five nonsynonymous substitutions in nsp3 (V430I, A616D), spike (D603G, Q617K), and M (L15F), as well as four synonymous changes at genome positions 10437, 16248, 27897, and 28803 (Fig. 1G, Supplementary Table 1). All nonsynonymous substitutions identified in the PRD0038 P4 were incorporated into the recombinant backbone, resulting in a genetically stable, trypsin-independent variant (rPRD0038). Notably, the spike D603G substitution in rPRD0038 occurs in a homologous position as the globally dominant early pandemic substitution D614G in SARS-CoV-2, which favored RBD-up conformations and increased virion infectivity^11–13,15^. Two derivative strains encoding either green fluorescent protein (GFP) or nano-luciferase (nLuc) reporter genes were recovered to facilitate quantitative assays (Fig. 1F, Extended Data Fig. 1).

### D603G and Q617K substitutions shift PRD0038 spike toward RBD-up conformation

**Extended Data Fig. 2:**
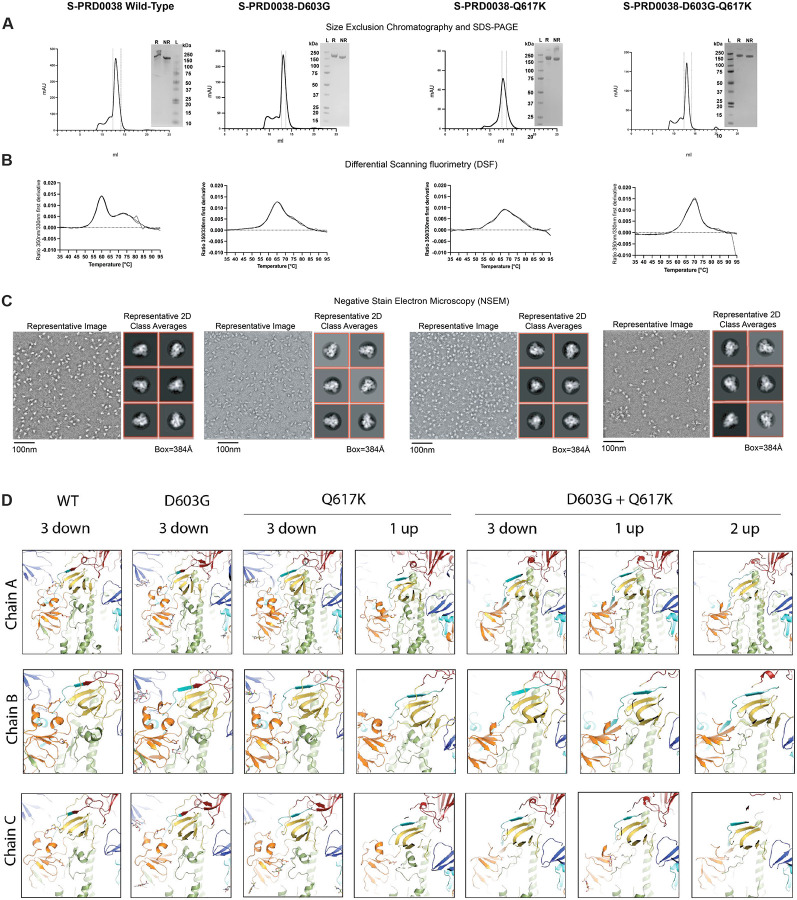
PRD0038 S protein purification and structural validation. **A**. Size exclusion chromatography chromatograms and SDS-PAGE analysis. The collected fractions are indicated by dashed lines on the chromatograms. **B.** Differential scanning fluorimetry (DSF) ratio 350/330 nm first derivative profiles. **C.** Negative Stain Electron Microscopy sample images and 2D classification for each sample. **D.** Shifts at the SD1/SD2 regions in each chain of PRD0038 S WT, D603G, Q617K, and D603G-Q617K structures. Structures are shown in cartoon view with the domains colored as in Fig. 3 and the view of Fig. 3B.

**Figure 2:**
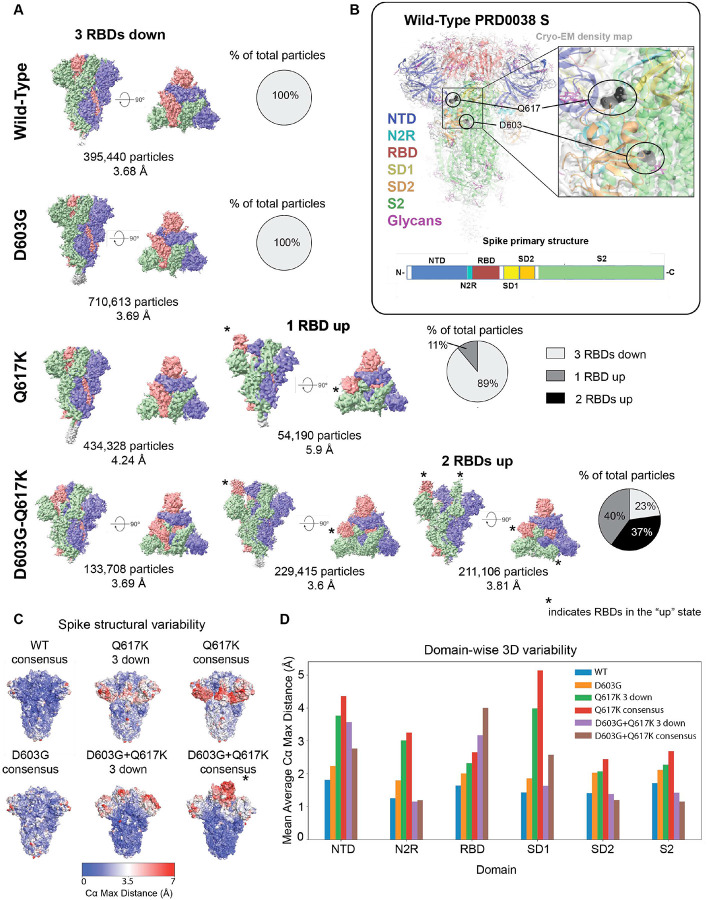
Conformational landscape of PRD0038-D603G-Q617K and single point mutations PRD0038-D603G and Q617K versus PRD0038-WT S proteins in cryo-EM analysis. **A.** 3 RBDs down, 1 RBD up, and 2 RBDs up structures solved from PRD0038-D603G-Q617K and PRD0038-WT cryo-EM datasets, with their resolutions and particle numbers in each population listed beneath. The 3 chains of the S protein are colored green, salmon, and purple. The percentage of total particles in each RBD up/down state is indicated by a pie chart for each dataset. **B**. PRD0038-WT S structure with electron density shown as a transparent gray surface, and the coordinates fit into the density colored by domain. Locations of residues 603 and 617 are shown as black spheres. **C.** Surface view sof PRD0038-WT 3-RBD-down S, PRD0038-D603G 3-RBD-down S, PRD0038-Q617K 3-RBD-down and consensus S, and PRD0038-D603G-Q617K 3-RBD-down and consensus S structures colored by Per-residue 3D variability quantified using C*α* maximum distance across the ensemble of 8 structures obtained from 3D variability analysis (3DVA). Blue corresponds to less variable and red corresponds to more variable. **D**. Domain-wise 3D variability with mean of the average C*α* maximum distance for each domain shown as bars. The * indicates RBDs in the “up” state throughout the figure.

To assess the structural impact of the D603G and Q617K substitutions on the rPRD0038 spike protein, we performed single particle cryo-EM analysis to determine structures of the recombinant PRD0038-D603G-Q617K and PRD0038 wild-type (WT) spike protein (S) ectodomains, as well as recombinant PRD0038-D603G and PRD0038-Q617K single point mutated protein ectodomains. The PRD0038-WT S ectodomain cryo-EM dataset yielded a 3.7 Å resolution map corresponding to a population of closed spikes with all three RBDs in the down conformation (Fig. 2A, Extended Data Fig. 2, Supplementary Fig.1). This is consistent with a previously published structure of PRD0038 S ectodomain with 5 pre-fusion stabilizing proline mutations, in which no spikes adopting the RBD-up conformation were observed (PDB: 8U29)^10^. In contrast, our PRD0038-D603G-Q617K dataset was dominated by open conformations, with ~40% 1-RBD-up (3.6 Å), ~37% 2-RBD-up (3.8 Å), and ~23% 3-RBD-down (3.6 Å) particles (Fig. 2A, Supplementary Fig.2), indicating that these substitutions shift spike conformational dynamics toward RBD-up states. The PRD0038-D603G point mutation alone yielded a single 3-RBD-down population resolved at 3.7 Å, similar to WT (Supplementary Fig.3), whereas the PRD0038-Q617K construct produced a small fraction of 1-RBD-up particles (~11%), resolved at 5.9 Å, but remained predominantly in the closed state (4.2 Å) (Fig. 2A, Supplementary Fig.4). These observations indicate that both substitutions are required and act cooperatively to optimize RBD-up transitions in the PRD0038 spike.

### Characterization of structural variability across the spike ectodomains

To further characterize the PRD0038 S protein conformational variability and assess the impact of the D603G and Q617K substitutions, we performed 3D variability analysis (3DVA). This approach quantifies structural heterogeneity within cryo-EM dataset particle populations and identifies regions of differential motion, providing insights into how specific substitutions influence spike dynamics.

For the PRD0038-Q617K spike, the 3-RBD-down and 1-RBD-up populations were combined to generate a 4.3 Å consensus map, in which all three RBDs were in the down position. The PRD0038-D603G-Q617K S 3-RBD-down, 1-RBD-up, and 2-RBD-up populations were combined to yield a 3.4 Å consensus map, in which one RBD appeared in the up position. Since the PRD0038-WT and PRD0038-D603G S datasets consisted exclusively of 3-RBD-down particles, these volumes were used directly for 3DVA (Supplementary Fig.5–8). For the PRD0038-Q617K and PRD0038-D603G-Q617K datasets, we performed 3DVA on both the 3-RBD-down and consensus particle populations.

Variability was quantified by fitting atomic models into the reconstructed sub-volumes and measuring the maximum Cα displacement for each residue across each ensemble, with values averaged across the three protomers, as described in the methods (Fig. 2C). Additionally, the average maximum distance was calculated for each S protein domain in the WT and mutant structures. Similar variability across the spike ectodomain was observed for the PRD0038-D603G and WT structures. In contrast, a greater increase in average maximum distance was observed across all spike domains in the Q617K structure, with average displacements increasing by ~1–3 Å. The combined D603G-Q617K substitutions produced domain-specific increases in variability localized to the NTD, RBD, and SD1, while variability in other domains remained comparable to WT in the consensus structure (Fig. 2D).

For the PRD0038-Q617K and PRD0038-D603G-Q617K datasets, similar patterns of variability were observed between the 3-RBD-down and consensus structures, with some differences (Fig. 2C and 2D). The PRD0038-Q617K consensus structure showed greater variability in all its domains compared to its 3-RBD-down structure (Fig. 2D). For the PRD0038-D603G-Q617K spike, the SD1 variability in the 3-RBD-down structure was comparable to WT and lower than the consensus structure, suggesting that the increased SD1 variability in the PRD0038-D603G-Q617K consensus structure was driven by the RBD-up states. The NTD variability was, by contrast, greater in the 3-RBD-down population than in the consensus population for the double mutant, suggesting that the RBD-up populations have a less variable NTD. Similar effects, albeit with smaller differences between the consensus and the 3-RBD-down populations, were observed for the SD2 and S2 for the PRD0038-D603G-Q617K spike (Figure 2D). This indicates that the RBD-up states in the PRD0038-D603G-Q617K spike are associated with lower variability than the 3-RBD-down state in regions outside those directly involved in RBD movement (SD1 and the RBD itself). Together, these results indicate that Q617K increases flexibility across the spike ectodomain, whereas the D603G and Q617K substitutions combined localize conformational variability to specific regions to stabilize RBD-up transitions.

### Destabilization of SD2 loops may drive RBD-up conformations in the PRD0038-D603G-Q617K spike.

**Extended Data Fig. 3:**
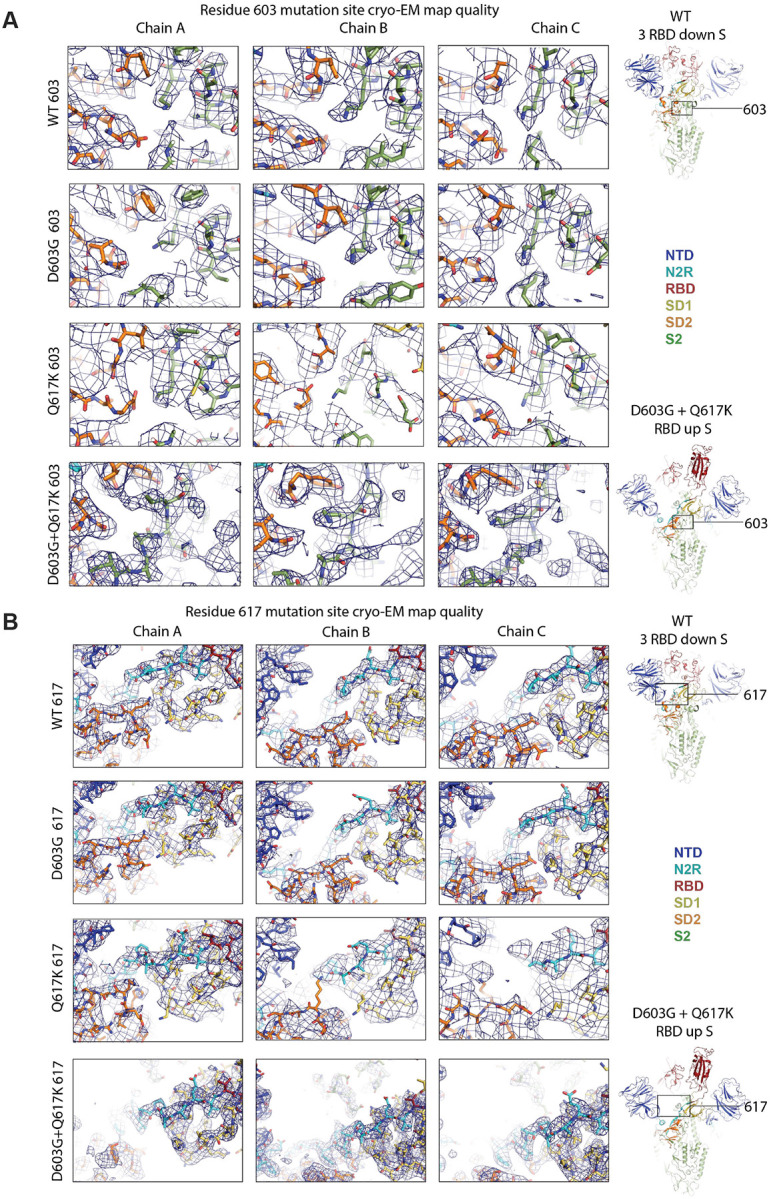
PRD0038 S Cryo-EM map quality at points of interest. **A**. Cryo-EM map shown in blue mesh with fit coordinates shown as sticks at the residue 603 site in chains A, B, and C for each consensus structure. **B.** Cryo-EM map shown in blue mesh with fit coordinates shown as sticks at the residue 603 site in chains A, B and C for each consensus structure.

**Figure 3:**
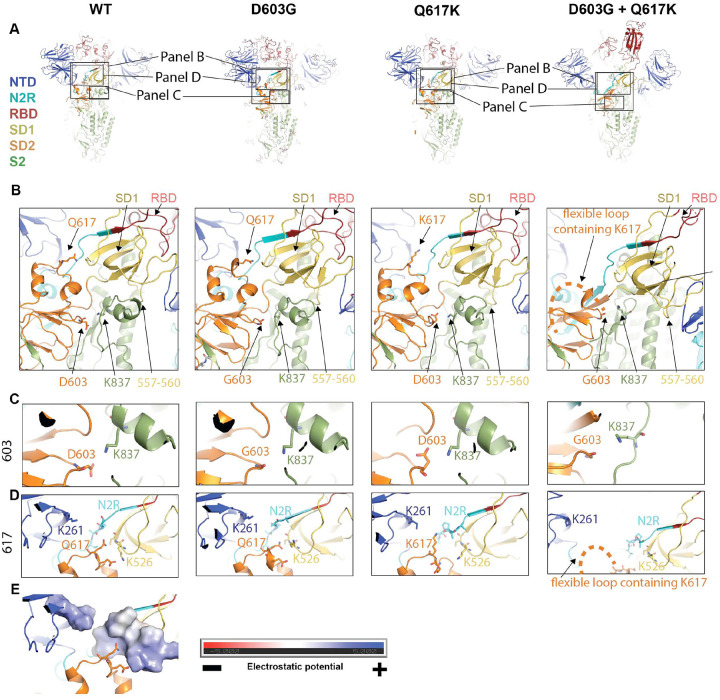
Conformational differences between PRD0038-D603G-Q617K, PRD0038-D603G, and PRD0038-Q617K versus PRD0038-WT S proteins. **A.** Global view of each consensus structure coordinate model indicating the regions detailed in panels B, C and D below. Domains are colored as indicated. **B.** Zoomed-in views of both mutation sites 603 and 617 on each structure. 3 Important interactions which are lost in the PRD0038-D603G-Q617K spike are indicated with arrows (1. Q617 to SD1/N2R interactions, 2. D603 to K837 interactions and 3. The interactions of the helix and loop containing K837 (stabilized by D603) with loop 557–560 of SD1). **C.** Details of the local interactions of residue 603 in the WT and the mutant spike structures. **D.** Details of the local interactions of residue 617 in the WT and the mutant spike structures. **E.** The same view as the left panel in 2D but with electrostatic potential shown in surface view for the regions of the NTD, SD1 and N2R that create hydrogen bonding interactions with Q617 of SD2, which are repelled upon the mutation to K at 617. In all panels of this figure, the four structures were aligned by S residues 908–922 to allow comparison of the same viewing positions across all structures.

The quality of the cryo-EM maps enabled atomic model building for the PRD0038-WT, PRD0038-D603G, PRD0038-Q617K, and PRD0038-D603G-Q617K S ectodomain consensus and subclasses, allowing direct structural comparison of substitution-induced conformational changes. Assessment of the local regions around the D603G and Q617K substitutions revealed potential mechanisms by which these substitutions destabilize the PRD0038 S ectodomain. The D603G substitution is in the SD2 subunit on its S2 subunit-facing side (Fig. 2B). In the PRD0038-WT structure, D603 forms a stabilizing interaction with K837 on a proximal ordered S2 helix near the base of SD1 (Fig. 3, left panels of 3A-C), supported by well-defined density in this region (Extended Data Fig. 3A). The ordered loop and helix spanning residues 812–837 stabilize interprotomer contacts that restrict SD1 mobility and limit its outward movement required for RBD-up transition. Interactions between R830 and D577 in the SD1 loop likely contribute to constraining SD1 motion. The D603G substitution disrupts this interaction, rendering the K837-containing helix disordered in the PRD0038-D603G-Q617K structure (Fig. 3C, right panel). These changes are consistent with SARS-CoV-2 D614G structure where interprotomer hydrogen bonding involving I834, Y837 or T859 of the S2 domain is disrupted by the mutation at 614, resulting in a more open S ectodomain^11–13,15,16^. In PRD0038, destabilization of the 812–837 loop and helix in the presence of D603G is associated with increased SD1 mobility; however, the D603G substitution alone does not induce the RBD-up conformation (Fig. 3C, middle panel), indicating that additional structural changes are required.

The Q617K substitution is located within the SD2 610–629 loop at the interface between SD2, SD1, and the NTD-to-RBD (N2R) region (Fig. 3B). In PRD0038-WT, this loop exhibits well-resolved density and engages an electropositive surface formed by residues 309–313 and 522–528 of the N2R/SD1 interface (Fig. 3E). In contrast, electron density for this loop is absent in the PRD0038-D603G-Q617K structure, consistent with increased local flexibility (Extended Data Fig. 3B). Substitution of Q617 with a positively charged K residue is predicted to introduce electrostatic repulsion at this interface, destabilizing SD1/N2R contacts and facilitating conformational transitions toward the RBD-up state (Fig. 3D, right panel).

Together, D603G and Q617K substitutions disrupt stabilizing SD1 intra-/inter-protomer interactions with SD2 and S2 at three key points (Fig. 3B, right panel), permitting outward displacement of SD1 and promoting transitions toward RBD-up conformations, exposing the receptor-binding interface, and potentially enhancing accessibility for *R. affinis* ACE2 engagement and cell entry.

### PRD0038 host tropism is restricted to regional bat and mammalian ACE2 orthologs

**Figure 4:**
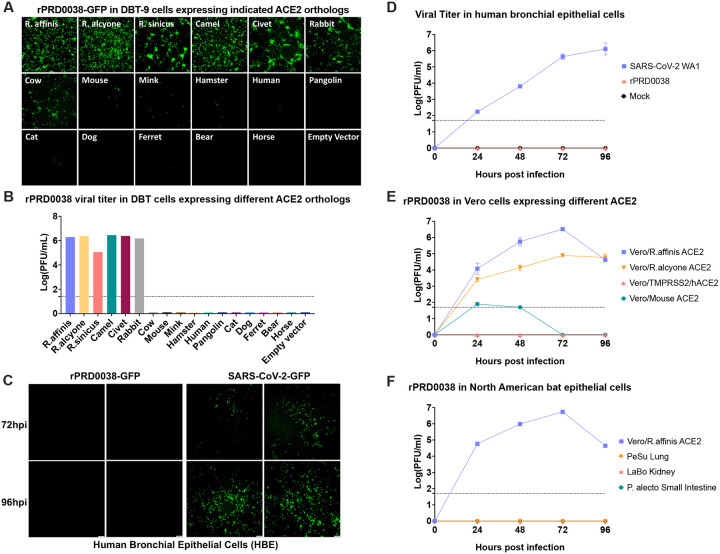
ACE2 receptor usage, Host range, and replication kinetics of rPRD0038. **A**. Fluorescence microscopy images of DBT-9 cells expressing indicated ACE2 orthologs or empty vector following infection with rPRD0038-GFP reporter virus at an MOI of 0.5. Images were taken 24h post-infection. **B.** rPRD0038 virus titer measured by plaque assay (PFU/mL) in DBT-9 cells expressing indicated ACE2 orthologs or empty vector at 3 days post-infection. Data are log10-transformed. Dotted line indicates the limit of detection (LOD) (50 PFU/mL). **C.** Fluorescence microscopy images of human bronchial epithelial cells (HBE) following infection with rPRD0038-GFP or SARS-CoV-2-GFP (WA1/2020) reporter viruses at an MOI of 1. Images were taken 72h and 96h post-infection. **D.** rPRD0038 and SARS-CoV-2 viral titers measured by plaque assay using apical washes collected from HBE cells at the indicated time points following infection at an MOI of 1. Data represent the log10(PFU/mL) mean ± s.e.m of two biological replicates. **E.** Multistep growth curve analysis (MOI = 0.5) of rPRD0038 in Vero cells expressing R. affinis, R. alcyone, or mouse ACE2, or Vero E6 cells overexpressing human TMPRSS2/ACE2. Data represent the mean ± s.e.m of a single experiment performed in triplicate. **F.** Multistep growth curve analysis (MOI = 0.5) of rPRD0038 in Vero/*R.affinis* ACE2 cells, or epithelial cells from North American bats, including *Perimyotis subflavus* lung cells, *Lasiurus borealis* kidney cells, and *Pteropus alecto* small intestine cells. Data represent the log10(PFU/mL) mean ± s.e.m of a single experiment performed in duplicate. Dotted line indicates the limit of detection (LOD) (50 PFU/mL).

To determine rPRD0038 host range and ACE2 receptor usage, we infected non-permissive DBT cells expressing ACE2 orthologs from diverse species. Fluorescence microscopy revealed robust replication in cells expressing *R. affinis*, *R. alcyone*, and *R. sinicus* bat ACE2, as well as *Paguma larvata/* (civet), *Camelus dromedarius* (camel), *Oryctolagus cuniculus* (rabbit), and *Bos taurus* (cow) ACE2 (Fig. 4A). All these species are present in Rwanda and across Africa, highlighting potential natural reservoirs for PRD0038. The human ACE2 receptor was not permissive for rPRD0038 entry. Plaque assays visualized 3 days post-infection in DBT-9 cells confirmed these patterns, with titers reaching ~10^6^ PFU/mL in *R. affinis*, *R. alcyone*, camel, civet, and rabbit ACE2 cells (Fig. 4B). Consistent with the lack of human ACE2 receptor usage observed in the DBT-9 cells, no rPRD0038 replication was detected in primary human bronchial epithelial cells at any time point by fluorescence microscopy or plaque assays. (Fig 4C and 4D). In contrast, SARS-CoV-2 (WA1/2020) replicated efficiently, reaching readily detectable levels by 24h post-infection (Fig 4C and 4D).

To further assess virus replication, we performed a multistep growth curve analysis (MOI = 0.5) in Vero cells expressing *R. affinis*, *R. alcyone*, human, or mouse ACE2. Maximal replication was detected in Vero/*R. affinis* ACE2 cells at 72h post-infection (~10^7^ PFU/mL) and lower titers in Vero/*R. alcyone* ACE2 cells (~10^5^ PFU/mL). Minimal virus replication titers were seen in Vero/mouse ACE2 cells (~10^2^ PFU/mL) and were undetectable by 72h post-infection. No replication was detected in Vero/human TMPRSS2/ACE2 at any time point (Fig. 4E).

To test the susceptibility of other phylogenetically and ecologically distinct bat epithelial cells to rPRD0038, we evaluated rPRD0038 replication in previously described^17^ immortalized epithelial cells from lungs of *Perimyotis subflavus*, kidneys of *Lasiurus borealis,* and Small intestines of *Pteropus alecto*, however, no detectable viral replication was observed (Fig. 4F).

Collectively, these results indicate that PRD0038 tropism is largely restricted to *Rhinolophus* bats and select mammals, present in Rwanda and sub-Saharan Africa. The data highlight potential regional spillover hosts while confirming that PRD0038 is not currently capable of infecting human cells.

### PRD0038 remains susceptible to approved antivirals and antibodies against conserved epitopes

**Figure 5:**
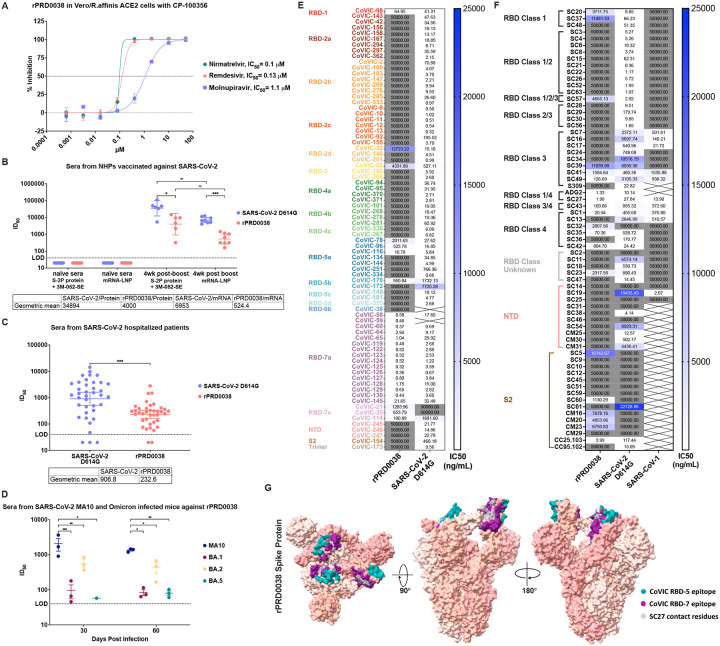
Cross-neutralizing antibody responses and susceptibility of PRD0038 to FDA-approved antivirals. **A**. Antiviral activity of remdesivir, molnupiravir, and nirmatrelvir against rPRD0038-nLuc reporter virus in Vero/*R.affinis* ACE2 cells in the presence of the P-glycoprotein inhibitor CP-100356. Quantitation of rPRD0038-nLuc replication measured by nLuc in technical duplicates. Cytotoxicity was measured in similarly treated but uninfected cultures via Cell-Titer-Glo assay. **B.** Live virus neutralization on serum collected from NHPs vaccinated with prefusion SARS-CoV-2 (WA1/2020) S-2P spike protein adjuvanted with 3M-052 or S-2P delivered by mRNA-LNP^21^ against rPRD0038-nLuc or SARS-CoV-2 D614G-nLuc reporter viruses on Vero/*R.affinis* ACE2 cells or Vero CCL-81 cells. Data are expressed as the geometric mean (95% CI) of half-maximal inhibitory dilution (ID_50_). Dotted line indicates the limit of detection (LOD). Values below the LOD are plotted at half the LOD. Data analysed by mixed effects analysis followed by Šidák’s multiple comparisons. *P = 0.0343 and ***P = 0.0004. **C.** Live virus neutralization using sera collected from hospitalized COVID19 patients (HVTN405, clinical trialNCT04403880) (n = 38) against rPRD0038-nLuc or SARS-CoV-2 D614G-nLuc reporter viruses on Vero/*R.affinis* ACE2 cells or Vero CCL-81 cells. Data are expressed as the geometric mean (95% CI) of half-maximal inhibitory dilution (ID_50_). Dotted line indicates the limit of detection (LOD). Values below the LOD are plotted at half the LOD. Data were analysed using a paired t-test. ***P = 0.0002 and ****P <0.0001. **D.** rPRD0038 Live virus neutralization using sera collected from mice at day 30 or day 60 post infection with MA10, BA.1 MA, BA.2 MA, or BA.5 MA^25^. Data were analysed by mixed effects analysis followed by Tukey’s multiple comparisons. *P = 0.0108, 0.0101, and 0.0456, **P = 0.0038 and 0.0100, and ***P = 0.0006. **E, F.** Heatmaps showing half-maximal inhibitory concentration (IC_50_) of the indicated monoclonal antibodies (mAbs) against rPRD0038-nLuc, SARS-CoV-2 D614G-nLuc, or SARS-CoV-nLuc (F). In **E**, CoVIC mAbs (CoVIC-DB; www.covic.lji.org) are colored according to epitope communities on the RBD, NTD, S2, or quaternary epitopes on full-length spike (Trimer) as classified by Schendel et al.^26^. In **F,** mAbs derived from SARS-CoV-2 infection, vaccination, or their combination are grouped based on their previous classification according to Barnes et al.’s four-quadrant classification^27–30^. X denotes assays not performed. **G.** Top, side, and back side views of the rPRD0038 spike structure, highlighting potent RBD mAbs target regions, colored by epitope communities. Green = epitope 5, Purple = epitope 7 and Gray = SC27 contact residues, which are within epitope 7.

Although PRD0038 is not capable of infecting human cells, virus emergence is often gradual through recombination and adaptation in intermediate hosts^8^. Given the presence of susceptible regional species and the conservation of key replication and structural elements with SARS-CoV-2, identifying effective countermeasures for PRD0038 is essential for pandemic preparedness. Therefore, we assessed the susceptibility of PRD0038 to three US Food and Drug Administration (FDA)-approved agents: the RNA-dependent RNA polymerase inhibitor remdesivir, the nucleoside analog molnupiravir, and the oral protease inhibitor nirmatrelvir. Antiviral activity was quantified using rPRD0038 nano-luciferase (nLuc) reporter virus in Vero cells expressing *R. affinis* ACE2. Assays were performed in the presence of the P-glycoprotein inhibitor CP-100356 to minimize drug efflux and better estimate intrinsic antiviral potency. rPRD0038 was highly sensitive to remdesivir, nirmatrelvir, and molnupiravir with half-maximal inhibitory concentration (IC_50_) values of 0.13 μM, 0.10 μM, and 1.1 μM, respectively (Fig. 5A). Assays performed in the absence of CP-100356 yielded higher IC_50_ values for remdesivir and nirmatrelvir (Extended Data Fig. 5A), likely due to limited metabolic conversion or reduced intracellular drug concentrations^18–20^.

To evaluate the impact of pre-existing SARS-CoV-2 immunity on PRD0038, live virus neutralization assays were conducted using sera from vaccinated non-human primates (NHPs), hospitalized COVID19 patients, and mice infected with ancestral or Omicron SARS-CoV-2 variants. rPRD0038 neutralization titers were compared to nLuc reporter SARS-CoV-2 D614G given its structural analogy to the rPRD0038. Sera from NHPs^21,22^ vaccinated with prefusion SARS-CoV-2 (WA1/2020) S-2P spike protein adjuvanted with 3M-052 potently neutralized rPRD0038 (mean ID_50_ = 4000), although significantly lower (~9 fold) than SARS-CoV2 D614G (mean ID_50_ = 34894; p = 0.0343) (Fig. 5B). Sera from NHPs immunized with the same S-2P antigen delivered by mRNA-LNP exhibited ~13-fold reduction in neutralization titers against rPRD0038 compared to SARS-CoV2 D614G, mean ID_50_ values of 524.4 and 6953, respectively (p = 0.0004) (Fig. 5B).

Cross-reactivity of sera from naturally infected COVID-19 hospitalized patients obtained from the NIH HIV Vaccine Trial Network (HVTN 405) early in the pandemic^23^ was assessed against rPRD0038 and compared to SARS-CoV-2 D614G. Mean neutralization titers against rPRD0038 were significantly lower than SARS-CoV-2 D614G (rPRD0038 mean ID_50_ = 232.6, SARS-CoV-2 D614G mean ID_50_ = 906.8; p = 0.0002), although most sera retained measurable cross-reactivity against rPRD0038 (Fig. 5C, Extended Data Fig. 5B).

Cross-neutralization activity of sera from mice infected with ancestral SARS-CoV-2 D614G MA10 was compared with sera from mice infected with Omicron variants (BA.1 MA, BA.2 MA, and BA.5 MA) collected at 30 and 60 days post-infection^24,25^.

Neutralization titers against rPRD0038 were significantly higher in MA10-infected mice than in mice infected with Omicron variants at both time points (MA10 ID_50_ = 2,070.8 and 1,347.2, BA.1 ID_50_ = 97 and 83.7, BA.2 ID_50_ = 572.6 and 435.2, BA.5 ID_50_ = 57.5 and 80.4; comparisons to MA10 p ≤ 0.0456) (Fig. 5D). These findings indicate that antibodies elicited by ancestral SARS-CoV-2 strains exhibit greater cross-neutralizing activity against zoonotic Sarbecoviruses than antibodies elicited by more recent SARS-CoV-2 variants of concern.

Next, we evaluated the cross-neutralization potential of a diverse panel of monoclonal antibodies (mAbs) synthesized or derived from SARS-CoV-2 immune response. The first panel included 77 therapeutic mAbs from the Coronavirus Immunotherapeutic Consortium (CoVIC) (CoVIC-DB; www.covic.lji.org)^26^. Of these mAbs, 26 neutralized rPRD0038 with IC_50_ titers ranging between 0.32 and 12723.2 ng/mL, and all of which bind the RBD of the spike protein (Fig. 5E). NTD, S2, and trimer-binding CoVIC antibodies did not display neutralizing activity against rPRD0038. CoVIC antibodies recognizing the soluble RBD cluster into seven primary epitope groups, RBD-1 through RBD-7^26^. The mAbs neutralizing rPRD0038 were clustered within groups RBD-1 and RBD-2, which bind the receptor-binding motif (RBM), RBD-5, which bind the outer face of the RBD and correspond to class-3 in the Barnes *et al*.’s four-quadrant classification^27^, and RBD-3 and RBD-7, which bind the inner face of the RBD and correspond to class-4 Barnes *et al*.’s four-quadrant classification. The most potent neutralizing antibodies were those in group 7a which binds a conserved epitope in the inner face of the RBD and has high ACE-2 blocking activity^26^. Our epitope sequence conservational analysis showed that RBD-6 and RBD-7 are the most conserved epitopes across sarbecoviruses from all different clades (Supplementary Data 2).

The second panel included 65 mAbs derived from SARS-CoV-2 infection, vaccination, or their combination^28^. Of these mAbs, 19 neutralized rPRD0038 with IC_50_ titers ranging between 1.98 and 16142.7 ng/mL (Fig. 5F). Among RBD-binding antibodies that neutralized rPRD0038, two corresponded to Barnes *et al*.’s class-1, four recognized epitopes between classes-1, −2, and −3, and seven were class-4 mAbs, including the previously described broadly neutralizing antibodies, SC1, SC27, and SC43^28^. Consistent with the CoVIC mAbs, the most potent rPRD0038 neutralizers targeted the highly conserved class 4 cryptic epitope, with SC27 achieving an IC_50_ of 1.98 ng/mL, comparable to the pan-Sarbecovirus antibody ADG-2 (IC_50_=1.33), which had lost neutralizing activity against SARS-CoV-2 variants beyond BA.1^29^. None of the NTD-binding mAbs were able to neutralize rPRD0038. Within the S2-targeting group, six mAbs neutralized rPRD0038, with CC25.103, which was derived from a hybrid response to SARS-CoV-2 infection and vaccination with Moderna mRNA-1273^30^, being the most potent (IC_50_ = 4 ng/mL) (Fig. 5F). Most of the antibodies that neutralized rPRD0038 were also cross-reactive to SARS-CoV, highlighting the breadth of mAbs that target conserved and structurally constrained regions of the spike. To visualize the regions targeted by these mAbs, we highlighted the target epitopes of the most potent mAbs in the spike structure of PRD0038-D603G-Q617K. These epitopes were CoVIC mAbs RBD-5 and RBD-7, with class 1/4 mAbs like SC27 binding residues within the CoVIC RBD-7 epitope (Fig. 5G).

Together, these findings indicate that while PRD0038 demonstrates substantial antigenic divergence from SARS-CoV and SARS-CoV-2 and limited susceptibility to most tested antibodies, certain broadly neutralizing RBD- and S2-directed mAbs retain activity. Moreover, currently available FDA-approved antiviral drugs remain effective against PRD0038, highlighting therapeutic options should a PRD0038-like virus emerge in humans.

### A bat ACE2-expressing mouse model for *in vivo* evaluation of PRD0038 countermeasures

Animal models are essential for evaluating countermeasures against emerging viruses. To assess rPRD0038 infectivity in laboratory mice, 10-week-old WT C57BL/6 mice, 64-week-old C57BL/6, ifnar−/− ifngr−/− double-knockout (DKO) mice, ifnar−/− ifnlr−/− DKO mice, or ifnlr−/− mice were inoculated intranasally with 1×10^5^ PFU of rPRD0038. Consistent with the lack of mouse ACE2 receptor usage observed *in vitro*, no infectious virus was detected in the lungs of these infected mice at day 2 post-infection (Extended Data Fig. 6A).

**Figure 6:**
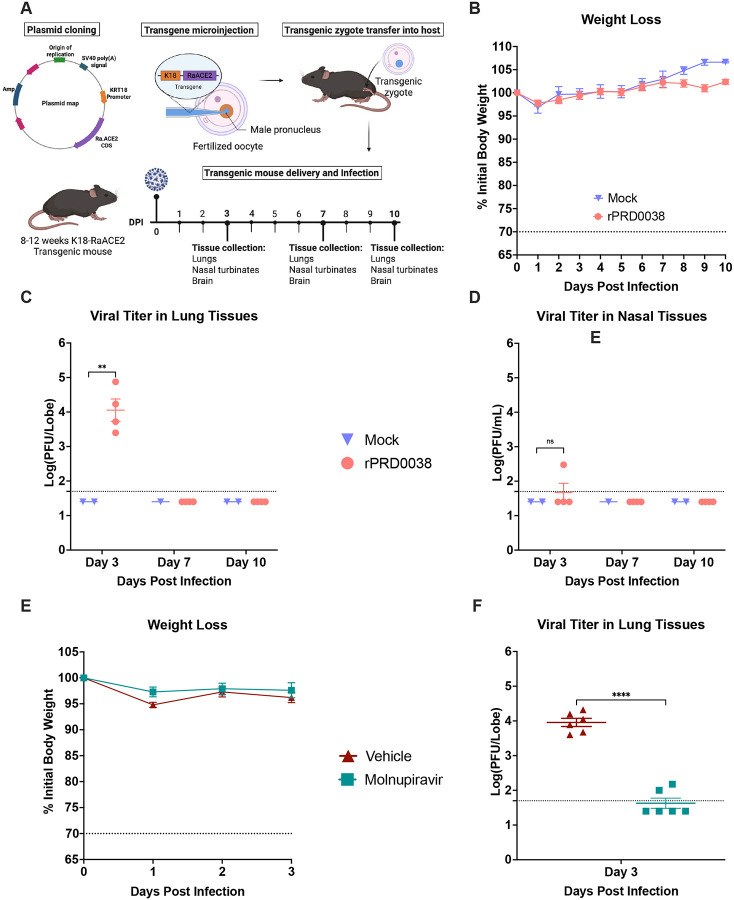
Development and use of an *R. affinis* ACE2-expressing mouse model for testing countermeasures against PRD0038 **A**. Schematic design of the cloning, generation, and infection of transgenic mice expressing the *R. affinis* ACE2 receptor (RaACE2) under the control of the cytokeratin-18 (K18) promoter. Created with BioRender.com **B.** Weight loss for K18-RaACE2 mice infected with 10^5^ PFU of rPRD0038 in 50μl PBS and monitored daily for 10 days. The dotted line represents weight-loss criteria for humane euthanasia. **C, D**. rPRD0038 viral titers measured by plaque assay following homogenization of lungs (C) and nasal turbinates (D). Dotted line indicates the limit of detection (LOD). Values below the LOD are plotted at half the LOD. The log10-transformed PFU/mL data were analysed by two-way ANOVA followed by Sidak’s multiple comparisons. In **C**, **P = 0.0037. **E.** Weight loss for K18-RaACE2 mice dosed with either vehicle or molnupiravir (EIDD-2801) at 200 mg/kg in 100μl by oral gavage 12 hours before intranasal infection with 10^5^ PFU rPRD0038 in 50μl PBS. Vehicle or drug was then administered every 12 hours for 3 days. The dotted line represents weight-loss criteria for humane euthanasia. **F**. rPRD0038 viral titers measured by plaque assay following homogenization of lungs at day 3 post-infection. Dotted line indicates the limit of detection (LOD). Values below the LOD are plotted at half the LOD. Log-transformed PFU/mL data were analysed by unpaired two-tailed t-test with Welch’s correction. ****P > 0.0001

To overcome this species barrier, we generated a transgenic mouse line expressing the *R. affinis* ACE2 receptor (RaACE2) under the control of the cytokeratin-18 (K18) promoter, analogous to the widely used K18-hACE2 model^31^. Using the published K18-hACE2 plasmid backbone (Addgene: Plasmid #149449), the human ACE2 coding sequence was replaced with the *R. affinis* ACE2 sequence (Fig. 6A). The resulting K18-RaACE2 construct was purified, linearized, and microinjected into C57BL/6J zygotes, which were subsequently implanted into pseudopregnant B6D2F1 females. Founder mice were identified by PCR screening, and three independent transgenic lines were established and propagated by backcrossing to wild-type C57BL/6 mice for at least one generation.

To assess *in vivo* replication, 8–12-week-old K18-RaACE2 mice were inoculated intranasally with 1×10^5^ PFU of rPRD0038 and monitored for 10 days. While rPRD0038 infection failed to elicit weight loss (Fig. 6B), high titer replicating virus was detected in the lungs of infected mice at day 3 post-infection (Fig. 6C). Viral titers in the nasal turbinates were detected in only one animal (Fig. 6D), and no infectious virus was detected in the brain at any time point (Extended Data Fig. 6B). Infectious virus was cleared from both the lungs and nasal turbinates by 7 dpi. No gross lung discoloration (GLD) was observed at any time.

Next, we evaluated the efficacy of a prophylactic antiviral treatment in this model. Eight- to twelve-week-old K18-RaACE2 mice received either the nucleoside analog molnupiravir (EIDD-2801) or vehicle control 12 hours prior to intranasal challenge with 1×10^5^ PFU of rPRD0038. Consistent with our previous data, there was no weight loss (Fig. 6E). However, at 3dpi, molnupiravir-treated mice exhibited a significant reduction in lung viral titers compared with vehicle-treated controls (Fig. 6F). These data underscore that receptor usage and pathogenicity are distinct properties, and that PRD0038 remains biologically constrained *in vivo*. Our data also demonstrates the utility of the K18-RaACE2 model for *in vivo* evaluation of countermeasures against PRD0038 and potentially other bat Sarbecoviruses.

## DISCUSSION

In this study, we report the successful recovery of a recombinant clade-3 African-lineage Sarbecovirus, rPRD0038. In the absence of other isolates, this virus provides a unique tool to evaluate the breadth and efficacy of existing vaccines, antivirals, and monoclonal antibodies. Our study defines the host range of rPRD0038 and highlights important species with potential spillover risk. Our data is consistent with earlier reports that PRD0038 would require important adaptive mutations in the RBD to promote infection of human hosts by zoonotic viruses^10^. Importantly, our study identifies effective small molecule inhibitors and therapeutic human monoclonals, providing strategies for disease control of emergent zoonotic viruses. These findings advance our understanding of Sarbecovirus diversity and inform surveillance and pandemic preparedness strategies.

Structural and functional characterization of rPRD0038 provides insight into Sarbecovirus biology and zoonotic potential. The wild-type PRD0038 spike, like many other zoonotic Sarbecoviruses, adopts a closed receptor-binding domain (RBD) conformation, limiting receptor accessibility and constraining viral infectivity^10^. Structural analyses of RaTG13, pangolin-CoV, and other bat coronaviruses have similarly shown that many prefusion spikes favor a compact, closed state, with RBDs in the down position, reducing ACE2 accessibility^32–35^. Cell culture recovery revealed D603G and Q617K variants that cooperatively shift the PRD0038 spike toward an RBD-up conformation, combined with increased receptor accessibility and infectivity resembling what was observed in SARS-CoV-2 D614G variants^11–13,15,16^. This conformational change, which did not alter receptor ortholog usage across the mammalian strains tested, also mirrors patterns seen in SARS-CoV and MERS-CoV, where RBD-up states facilitate receptor engagement and modulate viral entry efficiency^36–39^. A P607S change was previously described in SARS-CoV adaptation from the palm civet strain SZ16 to subsequent human epidemic strains^40^. This residue localizes to SD2, adjacent to a conserved conformational control region that may contribute to RBD-up movement^36,41^. Notably, the homologous position in SARS-CoV-2, P621, has been found substituted to serine in Omicron-derived BA.2.86 spike, where cryo-EM studies revealed ordering of the 620–640 segment and altered conformational behavior that may impact RBD accessibility^42^. These observations implicate this conserved SD2-proximal module as a recurrent site of evolutionary “fine-tuning” of spike conformational landscapes across Sarbecoviruses^41^. Additional conserved residues in this region, such as I621/V635 and A590/T604 in SARS-CoV versus SARS-CoV-2 alignments, may also modulate RBD opening and warrant systematic investigation, with implications for improved receptor binding, viral entry, and infectivity. These findings underscore the predictive value of structural characterization in assessing emergence risk and informing targeted surveillance of high-risk Sarbecovirus lineages in animal reservoirs.

Our host range analysis demonstrates that rPRD0038 does not efficiently engage the human ACE2 but uses ACE2 orthologs for entry from *Rhinolophus* bats as well as select mammalian hosts, including civet (*Paguma larvata*), camel (*Camelus dromedarius*), rabbit (*Oryctolagus cuniculus*), and cow (*Bos taurus*). This restricted receptor usage provides an added biosafety advantage for the use of this system in antiviral and immunological studies. *Rhinolophus* bats are widespread in African forests and peri-urban habitats and have been implicated as natural reservoirs for diverse Sarbecoviruses^43–46^. African civets are abundant and widespread; a concern as civets historically played a central role in the emergence of SARS-CoV 2003 by bridging bat-to-human transmission^43,47–50^. Camel ACE2 supports rPRD0038 entry *in vitro* as well. While natural Sarbecovirus infections in camels have not been reported, their established role as reservoirs and amplifiers of MERS-CoV highlights the importance of continued population-level surveillance^51–53^. Similarly, ACE2 from rabbits and cows, which are abundant and in close contact with humans, support rPRD0038 entry, but remain unstudied in natural contexts. By contrast, cells from geographically distinct bats did not support productive replication, likely due to divergence in ACE2, insufficient receptor expression, and/or the absence of activating host proteases^54^. These findings underscore the need to integrate ecological context, ACE2 compatibility, and systematic surveillance at the human–animal interface to evaluate zoonotic risk of emerging Sarbecoviruses^55,56^.

rPRD0038 remains broadly susceptible to existing antivirals that target conserved coronavirus replication machinery, consistent with other findings on the durability of these small-molecule countermeasures against divergent Sarbecoviruses^57,58^. Our data further indicate that pre-existing immunity to SARS-CoV-2 meaningfully shapes immune recognition of divergent Sarbecoviruses. Sera from mice infected with Omicron variants had diminished cross-neutralizing activity compared to ancestral SARS-CoV-2, indicating that ongoing SARS-CoV-2 viral evolution may gradually narrow population-level immunity to divergent zoonotic Sarbecoviruses, providing a potential pathway to human emergence^59,60^.

Antibody-mediated neutralization of rPRD0038 was strongly influenced by epitope conservation and spike conformational dynamics. Neutralizing activity was largely restricted to antibodies targeting conserved inner-face RBD epitopes and select S2 regions, consistent with observations across SARS-CoV, SARS-CoV-2, and related bat Sarbecoviruses^29,30,61^. Potent RBD class-4/RBD-7 antibodies identified in our study target cryptic epitopes that are largely inaccessible in the RBD-down conformation and require transient or stabilized RBD-up states for binding^26–28^. Our epitope conservation analysis identified RBD-6 and RBD-7 as the most conserved mAb targets across sarbecoviruses. Although limited data precluded functional assessment of RBD-6, all RBD-7–targeting monoclonal antibodies exhibited neutralizing activity against rPRD0038. Despite substantial sequence divergence within epitope 5, a subset of epitope 5a antibodies retained neutralizing activity, consistent with partial conservation of key residues within this region. For rPRD0038, the D603 substitution we identified promotes increased RBD-up occupancy, exposing a conserved epitope that would otherwise remain shielded. These findings reveal a fundamental evolutionary trade-off in which conformational shifts toward RBD-up states may facilitate host adaptation while simultaneously increasing susceptibility to pre-existing immune responses^62,63^.

Although escape mutations within conserved epitopes are possible, they often impose substantial fitness costs and require compensatory changes to maintain spike stability, fusion efficiency, or replication competence^7,29^. Such constraints may help explain the sustained activity of broadly neutralizing antibodies and replication-targeting antivirals across divergent sarbecoviruses and underscore the importance of layered countermeasures rather than reliance on a single therapeutic target. However, the preclinical evaluation of such countermeasures remains limited for sarbecoviruses with restricted host tropism. Several zoonotic sarbecoviruses, including BtKY72, WIV1, GX-PCoV, BANAL viruses, and other SARS-CoV- and SARS-CoV-2-related viruses utilize *R. affinis* ACE2 while exhibiting limited compatibility with mouse ACE2, restricting their study in conventional mouse models^7,64^. The K18-RaACE2 mouse model described here provides a new receptor-matched platform for evaluating viral pathogenesis and countermeasure efficacy against emerging sarbecoviruses with limited host range.

Our study integrates structural, molecular, and immunological methods to characterize PRD0038 and its zoonotic landscape. The conserved impact of RBD-up conformations on viral entry, the identification of mammalian species that may serve as spillover hosts, and the demonstrated efficacy of existing therapeutics together inform targeted surveillance, pandemic preparedness, and risk mitigation strategies. This work must be framed within a One Health context, recognizing that the interfaces between wildlife, domestic animals, and humans are dynamic and shaped by land use, agricultural practices, and urban expansion^65^. Proactive investments in such integrated studies will not only advance understanding of emerging viruses but also enable the rational development of broadly protective countermeasures. In this way, studies such as ours can inform policy and global health priorities, ensuring that the identification of novel Sarbecoviruses translates into actionable strategies to reduce the risk of future pandemics.

## METHODS

### Ethics and biosafety

Synthetic reconstruction of the authentic, wild-type PRD0038 virus was based on its published sequence and undertaken with the approval of the Institutional Biosafety Committee of the University of North Carolina at Chapel Hill (Application no. 99994). Incorporation of naturally acquired substitutions into the recombinant backbone was approved by the Institutional Biosafety Committee of the University of North Carolina at Chapel Hill (Application no. 163083). All animal work was approved by Institutional Animal Care and Use Committee at University of North Carolina at Chapel Hill according to guidelines outlined by the Association for the Assessment and Accreditation of Laboratory Animal Care and the US Department of Agriculture. All work was performed with approved standard operating procedures and safety conditions at biosafety level 3, with personnel wearing full-body personal protective equipment, including Tyvek suits, Tyvek aprons, booties, double gloves, hoods, and HEPA-filtered powered air-purifying respirators (PAPRs). Our institutional BSL3 facilities have been designed to conform to the safety requirements recommended by Biosafety in Microbiological and Biomedical Laboratories (BMBL), the US Department of Health and Human Services, the Public Health Service, the Centers for Disease Control and Prevention (CDC), and the National Institutes of Health (NIH). Laboratory safety plans have been submitted, and the facility has been approved for use by the UNC Department of Environmental Health and Safety (EHS) and the CDC.

### Phylogenetic and Sequence Similarity Analysis and Visualization

Genomic and protein sequences were aligned using Geneious Prime (v.2024.0). The resulting multiple sequence alignments were used to construct phylogenetic trees using FigTree (version 1.4.5). Trees were visualized and edited using FigTree with branch lengths scaled to substitutions per site. Major clades and bootstrap support values were annotated for clarity.

Sequence similarity plots (SimPlots) were generated to assess genomic or protein sequence homology across multiple strains or variants. Multiple sequence alignments were first performed using Geneious Prime (v.2024.0) MAFFT alignment. The aligned sequences were analysed using a Python-based implementation of SimPlot (SimPlot++), which computes pairwise percentage identity within a sliding window (Kimura two-parameter model, a window size of 1,000 base pairs, a step size of 100 base pairs, and a Gap/Strip parameter). Plots were generated using the Matplotlib library, with sequences color-coded for clarity.

### Cells and transfections

**Extended Data Fig. 4:**
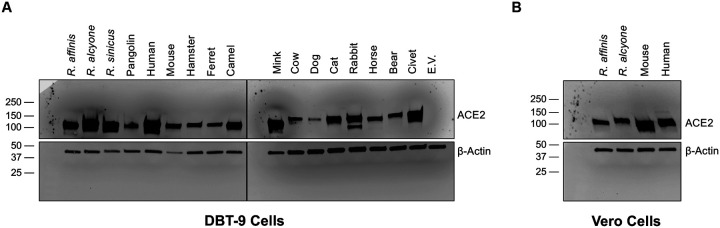
Western blot analysis of ACE2 orthologs in DBT-9 or Vero CCL-81 cells. **A**. Western blot analysis of ACE2 ortholog or empty vector (E.V.) expression from 17 mammalian species in DBT-9 cells. **B.** Western blot analysis of *R. affinis*, *R. alcyone*, mouse, or human ACE2 expression in Vero CCL-81 cells.

Vero CCL-81 cells (ATCC^®^ CCL-81^™^) and Vero C1008 cells (ATCC CRL-1586) were maintained in Dulbecco’s modified Eagle’s medium (DMEM; Gibco), 10% fetal bovine serum (FBS, Hyclone), and 1× antibiotic–antimycotic (Gibco). Mouse Delayed Brain Tumor cells (DBT-9) were previously clonally derived in our laboratory, maintained in DMEM, 10% fetal clone II serum (FCII, Hyclone), and 1× antibiotic–antimycotic. Cells were confirmed to be negative for mycoplasma contamination. For ACE2 receptor usage, bat *R. alcyone* (GenBank, ALJ94035), bat *R. sinicus* (GenBank, QMQ39213.1), mouse (GenBank, NP_001123985.1), and mink (GenBank, XP_044091952) ACE2 were cloned into a mammalian expression vector pcDNA3.1. These ACE2 vectors or empty pcDNA3.1 were then transfected into DBT-9 using Lipofectamine 2000 (Invitrogen) as previously described^66^. ACE2 orthologs including bat *R. affinis* (GenBank, QMQ39234.1), camel (GenBank, XP_010991717.1), civet (GenBank, AAX63775.1), rabbit (XP_002719891.1), cow (GenBank, AAX46514.1), hamster (GenBank, XM_005074209.3), human (GenBank, Q9BYF1.2), pangolin (GenBank, XP_017505752.1), cat (GenBank, BAE72461.1), dog (GenBank, NP_001158732.1), ferret (GenBank, NP_001297119.1), bear (GenBank, XP_044247820.1), and horse (GenBank, XP_001490241.2), were expressed in DBT-9 cells using the Sleeping Beauty Transposon System as previously described^14,67^. *R. affinis*, *R. Alcyone*, and mouse ACE2s were also expressed in Vero CCL-81 cells using the Sleeping Beauty Transposon System. Vero E6 cells over-expressing human TMPRSS2/ACE2 were a gift from Adrian Creanga and Barney Graham at the Vaccine Research Center (NIAID, Bethesda, MD). Puromycin (Gibco) was used as a selection antibody. Uniform ACE2 expression in these cells was confirmed by western blot analysis using a polyclonal ACE2 antiserum (Extended Data Fig. 4).

Primary human bronchial epithelial (HBE) cells were purchased from the Marsico Lung Institute Airway BioCore at the University of North Carolina at Chapel Hill. HBE cells were obtained from airway specimens resected from patients undergoing surgery under University of North Carolina Institutional Review Board-approved protocols (IRB no. 03–1396) as previously described^68^. Briefly, cells were differentiated at an air–liquid interface (ALI) for 6–8 weeks to produce well-differentiated, polarized cultures that recapitulate the pseudostratified mucociliary epithelium of the human airway.

### Generation of molecular cDNA clones and recovery of PRD0038 recombinant viruses

The full virus genome of PRD0038 (GenBank, accession no. MT726045.1) was separated into eight fragments and individually cloned into different plasmids that were chemically synthesized by Genescript with the incorporation of a T7-promoter sequence into the 5’-end of fragment A and a 25 nt poly-A tail into the 3’-end of the fragment H. Each fragment was verified by Sanger sequencing. To enhance the efficiency of recovering PRD0038 in the cell culture, an sgRNA-N construct encoding a 5’ leader sequence, an N gene, a 3’ UTR and a 25 nt poly-A tail was assembled downstream of a T7 promoter.

To recover the full-length virus, cDNA fragments were digested with appropriate restriction enzymes, resolved in 1% agarose gels, excised, and purified using a QIAquick Gel Extraction kit (Qiagen). A full-length genomic cDNA was obtained by ligating eight fragments in an equal molar ratio using T4 DNA ligase (NEB). The full-length PRD0038 genomic RNA or sgRNA-N was synthesized using the T7 mMESSAGE T7 transcription kit (Thermo Fisher) at 32 °C for 4 h. The RNA transcripts were then mixed and electroporated into Vero cells expressing *R. affinis* ACE2 at 450 V and 50 μF using a Gene Pulser II electroporator (Bio-Rad). Four rounds of enrichment were performed by culturing on fresh vero/*R.affinis* ACE2 cells in the presence of 5 μg/mL trypsin (Gibco).

Following whole genome next generation sequencing, the rPRD0038 quasispecies variant containing nonsynanomous substitutions was recovered by site-directed mutagenesis of the corresponding plasmids. The full-length rPRD0038 was then recovered by ligating eight fragments in equal molar ratios, synthesizing the genomic RNA, and electroporating into Vero cells expressing *R. affinis* ACE2. Two derivative reporter viruses containing a GFP or an nLuc gene were generated by replacing the ORF7a gene with the reporter genes as in generation of the SARS-CoV-2 infectious clone (PMIC 32526206). rPRD0038 virus stocks were produced on Vero/R.affinis ACE2 cells and titrated via plaque assay. In brief, virus was serially diluted and inoculated onto confluent monolayers of Vero/*R.affinis* ACE2 cells, followed by agarose overlay. Plaques were visualized 3 days post-infection by staining with neutral red dye.

### Whole Genome Sequencing

Viral RNA was extracted from virus stocks using TRIzol LS (Thermo Fisher Scientific) according to the manufacturer's instructions. Total RNA (100 ng) was used as input for the Illumina Stranded Total RNA Prep with Ribo-Zero Plus kit (Illumina). Libraries were prepared according to the manufacturer's protocol and sequenced on an Illumina MiSeq instrument using a v3 150-cycle kit, generating a minimum of 500,000 reads per sample. Sequencing reads were mapped to the corresponding full-length viral reference genome using the BBMap aligner (v38.84) in Geneious Prime (v2025.2.2; Biomatters). Single nucleotide variants (SNVs) were identified using the Geneious Prime Find Variations/SNPs function with a minimum variant frequency threshold of 0.02 and a minimum read coverage of 100.

### Lollipop Plot Generation

Mutation or variant distributions of rPRD0038 as identified by whole genome sequencing were visualized using lollipop plots to indicate the frequency and genomic position of changes. Python 3.8+ and Matplotlib were used for plotting. For each mutation, a vertical stem was drawn from the baseline to the observed frequency, with a marker at the top indicating the exact value. When multiple groups or conditions were compared, distinct colors and markers were used to represent each group, enabling direct visual comparison. Data were processed and formatted in pandas dataframes prior to plotting. All scripts and plotting code were implemented in Python 3.12 on macOS and are available upon request. Figures were exported in high-resolution PNG or PDF formats suitable for publication.

### Spike purification

The plasmids used for spike purification were obtained by gene synthesis and overlapping PCR to obtain the S-PRD0038-WT construct followed by site-directed mutagenesis to obtain the S-PRD0038-D603G-Q617K construct performed by GeneImmune Biotechnology (Rockville, MD) with quality controls. The WT PRD0038 ectodomain sequence as well as S-PRD0038 D603 Q617K ectodomain sequence were cloned into the plasmids. The constructs are attached to a C-terminal T4 fibritin trimerization motif, a C-terminal HRV3C protease cleavage site, a TwinStrepTag and an 8XHisTag, sequentially, and incorporated into pαH vector. The coding region was codon optimized for mammalian expression and incorporated into a pαH vector.

### S protein expression and purification

Plasmids encoding the S protein ectodomains were transfected into Gibco FreeStyle 293-F cells (embryonal, human kidney) following the manufacturer’s recommendations. Supernatant was harvested 6 days post transfection and filtered through a 0.22 μm filter. StrepTactin resin (IBA LifeSciences) was used to purify the S ectodomains, followed by size exclusion chromatography (SEC) on a Superose 6 10/300 GL Increase column (Cytiva, MA) equilibrated in 2 mM Tris, pH 8.0, 200 mM NaCl, 0.02% NaN_3_. SEC fractions containing the S protein were combined and concentrated (Extended Data Fig. 2A). All purification steps were performed at room temperature within a single day. Protein quality was assessed by using NuPage 4–12% (Invitrogen, CA) for SDS-PAGE. Absorbance at 280 nm was measured on a Thermo Scientific NanoDrop OneC UV-Vis Spectrophotometer to calculate S concentration. The final products were flash-frozen in liquid nitrogen and stored at −80°C in single-use aliquots for future use. Aliquots were thawed at 37 °C for 20 minutes before use.

### Differential Scanning Fluorimetry

DSF assays were performed using Tycho NT.6 (NanoTemper Technologies). S proteins were diluted to approximatively 0.124 mg/mL in Phosphate buffered saline PBS). Intrinsic fluorescence was measured at 330 nm and 350 nm while the sample was heated from 35 to 95°C at a rate of 30°C/min. The ratio of fluorescence (350/330 nm) and inflection temperatures (Ti) were calculated using the inbuilt software in the Tycho NT. 6 (Extended Data Fig. 2B).

### Negative-staining electron microscopy (NSEM)

A frozen aliquot from −80 °C was thawed at 37 °C in Al block for 20 min. Sample was then diluted to 30 μg/ml with 0.02 g/dl Ruthenium Red in HBS (20 mM HEPES, 150 mM NaCl pH 7.4) buffer containing 8mM glutaraldehyde. After 5 min incubation, glutaraldehyde was quenched by adding sufficient 1 M Tris, pH 7.4, to give 80 mM final Tris concentration and incubated for 5 min. Quenched sample was applied to a glow-discharged carbon-coated EM grid for 10–12 second, blotted, consecutively rinsed with 2 drops of 1/20X HBS, and stained with 2 g/dL uranyl formate for 1 min, blotted and air-dried. Grids were examined on a Philips EM420 electron microscope operating at 120 kV and nominal magnification of49,000x, and 24 images were collected on a 76 Mpix CCD camera at 2.4 Å/pixel. Images were analysed by 2D class averages using standard protocols with Relion 3.07 (Extended Data Fig. 2C).

### Cryo-EM sample preparation and data collection

The S proteins in 2 mM Tris, pH 8.0, 200 mM NaCl, 0.02% NaN_3_ were removed from the −80° C freezer and thawed at 37° C for 20 minutes. The S proteins were diluted to 2 mg/ml and 0.5% glycerol was added just before freezing. The final buffer contained 2 mM Tris, pH 8.0, 200 mM NaCl, 0.02% NaN_3_, 0.5% glycerol. Using a PELCO easiGlow^™^ Glow Discharge Cleaning System, a QuantiFoil Cu300 R1.2/1.3 grid (Electron Microscopy Sciences, PA) was glow discharged at 15 mA for 15 s with 10 s hold time. A 3 μl drop of each sample was applied on the grid. One piece of filter paper was utilized for blotting (2.5 s) after 30 s incubation at 22 °C and 95% humidity. A Leica EM GP2 plunge freezer (Leica Microsystems) was used for blotting and plunge freezing in liquid ethane. The grids were stored in liquid nitrogen until data collection. A Titan Krios (Thermo Fisher) TEM was used for data collection for the S-PRD0038 D603G Q617K Spike and a Thermo Fisher Tundra TEM was used for data collection on the S-PRD0038 WT Spike (Supplementary Table 2).

### Cryo-EM data processing

Particles images were picked from micrograph images by blob picker in cryoSPARC8 following CTF estimation and patch CTF estimation. 2D classification was performed and classes that resembled Spike trimers were selected. Particles making up selected 2D classes were used for the generation of an *Ab initio* volume through *ab initio* refinement. This job filtered some particles into trash classes while making one “good” *ab initio* volume. Several rounds of heterogeneous refinement were used to further remove bad particles. A single consensus volume was generated using all good particles from each of the two datasets through a final round of non-uniform refinement.

The consensus particles were input into a 3D classification job which generated 10 volumes. For the S-PRD0038 WT dataset, all the volumes had the 3 RBDs in the down state, so no new structures were generated. For the S-PRD0038 D603G Q617K dataset, volumes with 3 RBDs down, 1 RBD up and 2 RBDs up were generated from the consensus particles. 3 RBD down, 1 RBD up and 2 RBD up were pooled together, and a final round of *ab initio* refinement followed by non-uniform refinement were performed on each. All refinements were done with C1 symmetry (Supplementary Fig. 1–4). ChimeraX9 was used to initially fit the refined volumes with PDB model ID 8U29. The relevant mutations were added to each model and S protein models were refined using Phenix10 real space refinement and manual refinement in Coot11 and ISOLDE12 within ChimeraX (Supplementary Table 2, Extended Data Fig. 3)

### 3D variability analysis

3DVA was performed using the 3D variability job in cryoSPARC^69^. Masks and particles from the final non-uniform refinement jobs for 3 down and 1 up particle populations for each S were input into the 3D variability job. The filter resolution was set to 6–7 and the number of modes to solve was set to 3. The output of the 3D variability job was input to a 3D variability display job in the cluster mode. The particles were separated by their principal components into 8 sub-populations and volumes were reconstructed from each set of particles. The 8 reconstructed volumes were then further refined in a non-uniform refinement job. The 8 sub-volumes were initially fit with the consensus structure model in ChimeraX which was further refined with ISOLDE in ChimeraX, Coot, and real-space refinement in Phenix.

### 3D variability analysis quantification

To quantify the variability for each residue across the 8 sub-volume models obtained from 3DVA, we measured the maximum distance moved for each residue CA across the 8 sub-volume models (from one extreme of the movement to the other for each residue CA). Using a python code generated using ChatGPT, Cα atom 3D coordinates for each residue in each sub-volume model were extracted into a table. The distances between each residue’s Cα coordinate in each sub-volume model to each other Cα for the equivalent residue in the other 7 sub-volume models were then measured using the same python code. The maximum distance for each residue Cα was then identified and a single value for the maximum variability (distance) in Å was then assigned to each residue. The variability was then plotted with residue number on the X axis and maximum variability in Å on the Y axis. This results in a single variability plot for each Chain of the S trimer for each 3 down and 1 up particle population analyzed through 3DVA (Supplementary Fig. 5–8). To quantify average variability of all residues in each domain, the average max distance for each residue in each domain was averaged together to obtain a bar chart with a single average max distance value for each S domain (Figure 2D).

### PRD0038 antiviral assay

The antiviral activity of remdesivir, molnupiravir, and nirmatrelvir against rPRD0038 was measured in Vero/*R.affinis* ACE2 cells that were plated at 20,000 cells per well in black-walled 96-well plates (Corning 3916). Cells were exposed to serial dilutions of compound and control compounds in infection medium (DMEM with 5% FBS) with or without P-glycoprotein efflux inhibitor at a fixed concentration (0.5 μM CP-100356) and incubated for 1 hour. rPRD0038-nLuc was then added at 800 PFU per well. At 24h post-infection, virus replication was measured by nLuc assay using a Promega Glomax plate reader (Promega). The IC50 value was defined in Graphpad Prism 10.6.0 (Graphpad) as the concentration at which a 50% reduction in the relative light units (RLU) was observed relative to the average of the virus-only control wells.

### Live-virus neutralization assay

Vero/R.affinis ACE2 cells (for rPRD0038) or Vero C1008 cells (for SARS-CoV-2 D614G) were plated at 20,000 cells per well in black-walled 96-well plates (Corning 3916) in DMEM (Gibco), 10% fetal bovine serum (Hyclone) and 1X antibiotic-antimycotic (A.A., Gibco). HIV Vaccine Trial Network (HVTN 405) sera originated from clinical trial NCT04403880. Serum samples were tested at a starting dilution of 1:40, and mAb samples were tested at a starting concentration of 30 to 0.1 μg/mL and were serially diluted 3-fold for up to eight dilution spots. Diluted antibodies and sera were then mixed with 800 PFU per well of rPRD0038-nLuc virus in infection medium (modified growth medium with 5% FBS), and the mixtures were incubated at 37 °C with 5% CO2 for 1h. Following incubation, growth media were removed cells and virus-antibody mixtures were added to the cells in duplicate. Virus-only controls were included in each plate, and all samples were run in duplicate. Following infection, plates were incubated at 37 °C with 5% CO2 for 28–30h. After the incubation, cells were lysed, and luciferase activity was measured via a Nano-Glo luciferase assay system (Promega) according to manufacturer specifications. Neutralization titers were defined as the sample dilution at which a 50% reduction in the relative light units (RLU) was observed relative to the average of the virus control wells.

### Mice and *in vivo* infections

The WT C57BL/6 mice were purchased from the Jackson Laboratories. The ifnar−/− ifngr−/− double-knockout (DKO) mice, ifnar−/− ifnlr−/− DKO mice, and ifnlr−/− mice were the generous gift of Helen M. Lazear (University of North Carolina at Chapel Hill).

The K18-RaACE2 mice were generated at The UNC Animal Models Core Facility (https://www.med.unc.edu/amc) under the direction of Dale Cowley. The K18-RaACE2 vector was cut with the restriction enzymes HpaI and XbaI generating a 6814 bp transgene fragment and a 2712 bp vector backbone fragment. The two fragments generated from the restriction digestion were separated on a 1% agarose gel (Apex General Purpose Agarose, Ultra Pure) in 1x TAE buffer. The 6814 bp transgene band was excised from the gel using a clean razor blade and purified using a modified version of the Qiagen gel extraction protocol. Briefly, the excised band was weighed to estimate volume and 3 volumes of QG buffer (Qiagen) were added to the tube. The tube was incubated for 10 minutes at 55°C with vortexing every two minutes. Once the agarose was dissolved in the QG buffer, the buffer was passed through EZ-10 spin columns (Bio Basic) using a Qiagen vacuum manifold, applying 250mg of agarose per column. Each column was washed with 750 μl of Wash Buffer (GenCatch), and then microcentrifuged for one minute at 18,213 × g. The transgene was eluted from the column with 50μl of buffer EB (Qiagen). The concentration was estimated by Nanodrop spectrophotometer (ThermoFisher) and a sample was analysed by agarose gel with band densitometry to quantitate for microinjection.

C57BL/6J zygotes were microinjected with the K18-RaACE2 transgene and injected embryos were implanted in recipient pseudopregnant B6D2F1 females. Sixteen resulting pups were screened by PCR for the presence of the transgene. Three positive founder (#26, 28 and 32, all males) animals were transferred for breeding and expression characterization.

Mice were infected intranasally under ketamine/xylazine anesthesia with 1×10^5^ PFU of rPRD0038 in 50μL of PBS. Body weight was monitored daily. At indicated timepoints, a subset of mice was euthanized by isoflurane overdose and were harvested for titer, RNA, and histopathology analyses. Titer and RNA samples were stored at −80°C, and histopathology samples at 4°C in 10% phosphate buffered formalin. Virus titers were determined via plaque assay as described above.

### *in vivo* antiviral treatment

To evaluate efficacy of molnupiravir *in vivo*, 8–12 weeks old K18-RaACE2 mice were dosed with either vehicle (10% PEG and 2.5% Cremophor RH 40 in water) or molnupiravir (EIDD-2801) at 200 mg/kg in 100 μl by oral gavage 12 hours before intranasal infection with 1 × 10^5^ PFU rPRD0038 in 50μl. Mice were anesthetized ketamine/xylazine before intranasal infection. Vehicle or drug was then administered every 12 hours for the remainder of the study.

## Extended Data

**Extended Data Fig.5:**
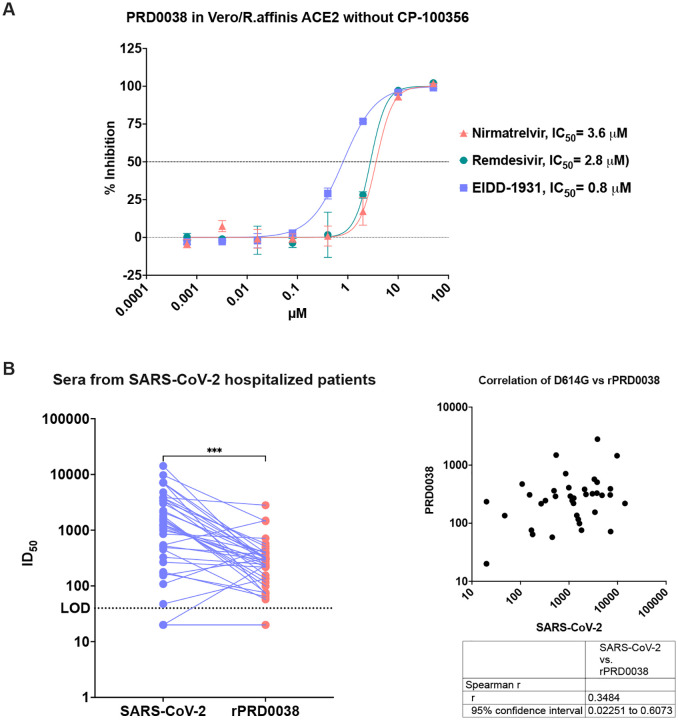
Susceptibility of PRD0038 to FDA-approved antivirals and preimmune sera. **A**. Antiviral activity of remdesivir, molnupiravir, and nirmatrelvir against rPRD0038-nLuc reporter virus in Vero/*R.affinis* ACE2 cells without the P-glycoprotein inhibitor CP-100356. Quantitation of rPRD0038-nLuc replication measured by nLuc in technical duplicates. **B.** Correlation analysis of the neutralization titers (ID50) against rPRD0038-nLuc and SARS-CoV-2 D614G-nLuc live viruses using sera collected from hospitalized COVID-19 patients (HVTN405, clinical trial NCT04403880). Left, paired ID50 values for individual sera against both viruses. Right, Spearman correlation analysis of ID50 values between viruses, with correlation coefficients (r) and 95% confidence intervals shown. ***P = 0.0002.

**Extended Data Fig.6:**
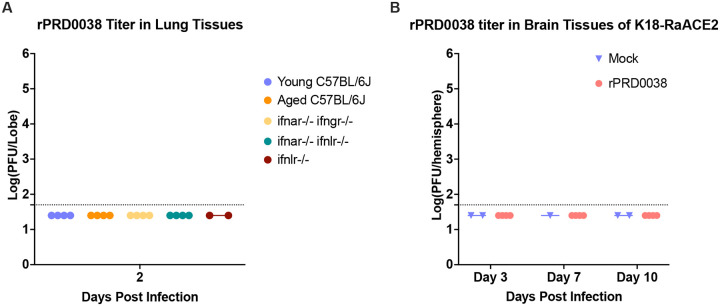
Absence of detectable rPRD0038 replication in different mouse models and brain tissues of K18-RaACE2 mice. **A**. PRD0038 viral titers measured by plaque assay following homogenization of lungs of 10-week-old WT C57BL/6 mice, 64-week-Old C57BL/6, ifnar−/− ifngr−/− double-knockout (DKO) mice, ifnar−/− ifnlr−/− DKO mice, or ifnlr−/− mice inoculated intranasally with 10^5^ PFU of rPRD0038. **B.** PRD0038 viral titers measured by plaque assay following homogenization of brain tissues of K18-RaACE2 mice inoculated intranasally with 10^5^ PFU of rPRD0038. The dotted line indicates the limit of detection (LOD). Values below the LOD are plotted at half the LOD.

## Supplementary Material

Supplementary Files

This is a list of supplementary files associated with this preprint. Click to download.

- 5.PRD0038SupplementaryData2mAbsepitopesconservationacrossSarbecoviruses.pptx
- 7.PRD0038SupplementaryTable2cryoemstats.xlsx
- 6.PRD0038SupplementaryTable1seq.xlsx
- 4.PRD0038SupplementaryData1CryoEM052426copy.pdf

## Data Availability

All data supporting the findings of this study are available within the paper and its Supplementary Information files. Cryo-EM maps and atomic coordinates have been deposited in the Electron Microscopy Data Bank (EMDB) and Protein Data Bank (PDB) and are currently on hold pending publication. rPRD0038 sequences generated in this study have been deposited to GenBank and will be released upon publication.
